# Chitosan-Alginate Nanoparticles as a Novel Drug Delivery System for Nifedipine

**Published:** 2008-09

**Authors:** Ping Li, Ya-Ni Dai, Jun-Ping Zhang, Ai-Qin Wang, Qin Wei

**Affiliations:** 1 *The Second Hospital of Lanzhou University, Lanzhou, China;*; 2 *Pharmacy College of Lanzhou University, Lanzhou, China;*; 3 *Lanzhou Institute of Chemical Physics, Chinese Academy of Sciences, Lanzhou, China*

**Keywords:** chitosan, nanoparticles, nifedipine, hydrogels, drug delivery system

## Abstract

Chitosan-alginate (CS/ALG) nanoparticles were prepared by ionotropic pre-gelation of an alginate core followed by chitosan polyelectrolyte complexation, nifedipine was chosen as a model drug. Morphology and structure characterization of nanoparticles were investigated by transmission electron microscope (TEM) and Fourier transform infrared spectra (FTIR), respectively. The diameter of the nanoparticles was about 20-50 nm, suitable for uptake within the gastrointestinal tract due to their nanosize range and mucoadhesive properties. A reversed-phase high-performance liquid chromatographic (HPLC) method has been developed and validated for the determination of nifedipine in nanoparticulate dosage forms. In addition, the delivery behavior of nifedipine from nanoparticles was studied. Nifedipine released from chitosan-alginate nanoparticles was 26.52% at pH1.5, 69.69% at pH6.8 and 56.50% at pH7.4 within 24hour. This suggests that the release of nifedipine from nanoparticles was pH-responsive. Quick release occurred in simulated intestinal fluid (SIF, pH6.8) and phosphate buffer solution (pH7.4), while the release was slow in simulated gastric fluid (SGF, pH1.5). The release profile was characterized by an initial burst effect in three media, followed by a continuous and controlled release phase, the drug release mechanism from polymer was due to Fickian diffusion.

## INTRODUCTION

One of the most attractive areas of research in drug delivery today is the design of nanosystems that are able to deliver drugs to the right place, at appropriate times and at the right dosage. Nanoparticulate delivery systems have the potential power to improve drug stability, increase the duration of the therapeutic effect and permit administration through enteral or parenteral administration, which may prevent or minimize the drug degradation and metabolism as well as cellular efflux (1-3).

Nanoparticles consisting of synthetic biodegradable polymers, natural biopolymers, lipids and polysaccharides have been developed and tested over the past decades. Recently, the idea of using nanoparticles made from natural biodegradable polymers to deliver drugs has provoked great interests. Among them, alginate and chitosan are very promising and have been widely exploited in pharmaceutical industry for controlling drug release (4, 5).

Alginate (ALG) is a water soluble linear polysaccharide extracted from brown sea weed and is composed of alternating blocks of 1-4 linked α-L-guluronic and β-D-mannuronic acid residues. ALG has been reported to be mucoadhesive, biodegradable, and biocompatible and has potential for numerous pharmaceutical and biomedical applications such as drug delivery system and cell encapsulation (6, 7). Alginate micro and nanoparticles can be obtained easily by inducing gelation with calcium ions (8, 9). Such easy-gelling property can be used to produce a pre-gel consisting of very small aggregates of gel particles, followed by the addition of an aqueous polycationic solution to make a polyelectrolyte complex coating (1). Poly-L-lysine (PLL), a cationic natural polymer, has been used to combine with ALG to prepare nanoparticles. However, PLL is toxic and immunogenic if injected. Recently, chitosan (CS) was selected as an alternative cationic polymer. Chitosan, a linear polysaccharide consisting of glucosamine and N-acetylglucosamine units, is biocompatible, biodegradable, and nontoxic in the application of peroral delivery of drugs (5). The addition of CS can not only endow nanoparticles positive surface charge, but also prolong the time that the active ingredients contact with the epithelium and enhance absorption via the para-cellular transport pathway through the tight junctions (10, 11).

Although many drugs (protein, insulin, DNA, cyclosporin A, etc.) (12-15) have been extensively investigated using natural polymeric carriers, the studies on the release of antihypertensive drugs are limited. The active substance of this investigation, nifedipine (NF), is an oral calcium-blocking agent used in the treatment of angina pectoris and hypertension; although it is absorbed almost completely, it displays a low bioavailability because of its short biological half-life with significant fluctuations in plasma concentrations (16) and its extensive first-pass metabolism (17). It was easily decomposed in light. Therefore, it is essential to prepare a controlled release formulation of NF and then CS/ALG nanoparticles were designed.

The objective of the present study was to evaluate the possibility of CS/ALG nanoparticles as carriers for nifedipine. The challenge was to entrap a hydrophobic molecule into hydrophilic nanoparticles formed by ionotropic pre-gelation of the positively charged polysaccharide chitosan and polyanion alginate. On the basis of previous work about chitosan hydrogels (18-20), chitosan-alginate nanoparticles were prepared by ionotropic pre-gelation of an alginate core followed by chitosan polyelectrolyte complexation. The morphology and infrared spectrum of nanoparticles, drug content of nanaparticles, the release properties of nanoparticles in simulated gastric fluid (SGF, pH1.5), simulated intestinal fluid (SIF, pH6.8) and phosphate buffer solution (pH7.4) were also studied.

## MATERIALS AND METHODS

### Materials

Chitosan (MW is 750000, degree of deacetylation is 80%) was acquired from Lanzhou Institute of Chemical Physics, the Chinese Academy of Sciences (Lanzhou, China). Sodium alginate of low viscosity (0.02 Pas) for a 1% solution at 20°C was purchased from Shanghai Chemical Co. Ltd (China), nifedipine (purity~99.7) was prepared in the laboratory(shanxi, China). All other chemicals and reagents used were of analytical grade.

### Preparation of Blank Chitosan-Alginate Nanoparticles

Both the sodium alginate and calcium chloride solutions were prepared by dissolving the chemicals in distilled water. The pH of the sodium alginate solution was adjusted to 5.1 using hydrochloric acid. Briefly, a known amount of chitosan was dissolved in 1% acetic acid solution and pH was modified to 5.4 using NaOH.

The method used to prepare the nanoparticles is a two-step method adapted from Rajaonarivony’s method of preparing alginate-poly-L-lysine nanoparticles (21). Aqueous calcium chloride (2 ml of 3.35 mg/ml) was added dropwise to 10 ml aqueous sodium alginate (3.0 mg/ml) while stirring for 30 min (XK97-2 two-way homoisothermy magnetic stirrer, 1200 rpm), and then 4 ml chitosan solution (0.8 mg/ml) was added into the resultant calcium alginate pre-gel and stirred for an additional 1 hour. The resultant opalescent suspension was equilibrated overnight to allow nanoparticles to form uniform particle size.

### Preparation of Nifedipine Loaded Chitosan-Alginate Nanoparticles

A constant volume (300 μl) nifedipine solution in a dehydrated alcohol/water mixture (1:1, 1.105 mg/ml) was incorporated into the calcium chloride solution, then the other processes were the same as the preparation of blank chitosan-alginate nanoparticles.

### Morphology and Structure Characterization of Nanoparticles

The morphology and particles size measurements of the nanoparticles were performed by transmission electron microscope (TEM) (JEOL, JEM-1230, Japan Ltd.). Nanoparticles separated from suspension were dried by a freeze dryer (FD-1, Beijing, China), their Fourier transform infrared spectra (FTIR) were taken with KBr pellets on using FW-4A pelleter on a FTIR spectrometer (Thermo Nicolet, NEXUS, TM, USA).

### Quantitative Analysis of Nifedipine

**Apparatus and Chromatographic Conditions.** Nifedipine was assayed by reversed-phase liquid chromatography (RP-HPLC) using a Agilent HPLC system (Agilent 1100, USA) equipped with UV detector and a ZORBAX SB-C_18_ (4.6 mm × 150 mm, 5μm). 20 μl samples were eluted with a mobile phase comprising 1% acetic acid solution (adjusted to pH3.31 with triethylamine)-methanol (30:70). The flow rate was 0.8ml/min and detection wavelength was 237 nm. Nifedipine was quantified by peak area measurement.

**Preparation of Standard Solutions.** Stock solutions of nifedipine were prepared by dissolving nifedipine in methanol at a concentration of 9 μg/ml. Nifedipine stock solution was further diluted with methanol to obtain the different working solutions. The standard solutions were prepared at concentrations of 0.18, 0.45, 1.8, 3.6, 4.5, 6.3 and 7.2 μg/ml.

**Preparation of Sample Solutions.** Accurately weighed samples (37-40 mg) were transferred into a conical flask, then nifedipine was extracted from chitosan-alginate nanoparticles by 50ml citric acid buffer solution (pH6.84, 0.4% Tween-80 v/v). Tween-80 was added into the media as a surfactant to increase the solubility of nifedipine, then the extract was centrifuged at 17,000 × g and 4°C for 30 min (SORALL^®^ Bioguge Stratos Ultracentrifuge), the supernatant was extracted by trichlormethane for three times (20, 20, 10 ml), and the residue was dissolved in methanol after evaporation of trichlormethane, the solution was filtrated by 0.45 μm millipore filter and determined by HPLC. All the experiments were carried out in triplicate.

### *In Vitro* Release of Nifedipine

To establish the nifedipine release profiles from nanoparticles at simulated gastric fluid (pH1.5), simulated intestinal fluid (pH6.8) and phosphated buffer solution (pH7.4). The NF-loaded nanoparticles were separated from the aqueous suspension by ultra-centrifugation at 17,000 × g and 4°C for 30 min (SORALL^®^ Bioguge Stratos Ultracentrifuge), the precipitates were dried by a freeze dryer. About 55-60 mg dried nanoparticles were placed into conical flask containing 75 ml dissolution medium and incubated at 37°C under stirring(120 rpm/min, ZRS-4 Intelligent Dissolution Tester, China), tween-80 (0.4%) was added into the media as a solubilizer. At appropriate intervals, 5 ml solution was taken and replaced by fresh medium, then it was centrifuged at 17,000 × g and 4°C for 30 min to eliminate possible insoluble polyions and the amount of nifedipine released from the nanoparticles was evaluated by HPLC as described above. The calibration curve was made using nonloaded NF nanoparticles as correction. All release tests were run in triplicate, and the mean value was reported.

### Release kinetics

The mathematical models, first-order *In [1-M_t_/M_∞_] = -kt*, Higuchi *M_t_/M_∞_ = kt^1/2^*, and zero-order *F(t)=kt* equations were fitted to individual dissolution data at two different time range with linear regression by SPSS 11.0 for Windows. The drug release mechanisms of chitosan-alginate nanoparticles were described by a semi-empirical equation.

## RESULTS

### Preparation of Chitosan-Alginate Nanoparticles

The chitosan-alginate nanoparticles are carried out at ambient temperature, preparation is simple, rapid, and reliable. CS/ALG nanoparticles are obtained spontaneously under very mild conditions.

The preparation of nifedipine loaded CS/ALG nanoparticles are relatively difficult. Because nifedipine is hydrophobic, these hydrophilic nanoparticles are used to encapsulate hydrophobic drug is not clear. A number of experiments had to be performed in order to determine the appropriate conditions for the incorporation of the hydrophobic nifedipine into the CS/ALG nanoparticles.

Ford *et al* (22) had previously shown that hydrophobic peptide cyclosporin A dissolved in water miscible organic solvents can be precipitated in an aqueous phase as discrete spherical particles. This precipitation process was also found to occur in our preparation. A successful entrapment was achieved by dissolving the hydrophobic nifedipine in a dehydrated alcohol/water mixture (1:1) prior to its incorporation into the calcium chloride, followed by the addition of the CS solution. In fact, following addition of the dehydrated alcohol/water nifedipine solution to a water phase, a Tyndall effect was observed, thereby forming a suspension of nanocrystals of nifedipine. Therefore, after incorporation of the CS to the calcium alginate pre-gel containing the nifedipine nanocrystals, calcium alginate pre-gel in the form of discrete nanoparticles simultaneously entrapping the nifedpine nanocrystals suspended in the medium. Hence, the final product consists of a suspension of CS/ALG nanoparticles containing nifedipine nanocrystals entrapped within the CS molecules.

In fact, calcium ions react with guluronic acid units on ALG to form an ‘egg-box’ structure. It is proposed that nanoparticles can be formed by enveloping the negatively charged calcium alginate complex in pre-gel state with cationic polymer, and the pre-gel state is essential to enable the ionic interactions between ALG, calcium, and cationic polymer to form nanoparticles (23). Based on previous single factor design and orthogonal experiment, the optimal mass ratio range of sodium alginate: CaCl_2_: chitosan to prepare nanoparticles was 30:6.7:3.2. This mass ratio ensured that the calcium alginate was maintained in the pre-gel phase and sufficient cationic polymer was present to form nanoparticles.

### Morphology and Structure Characterization of Nanoparticles

**Figure 1:**
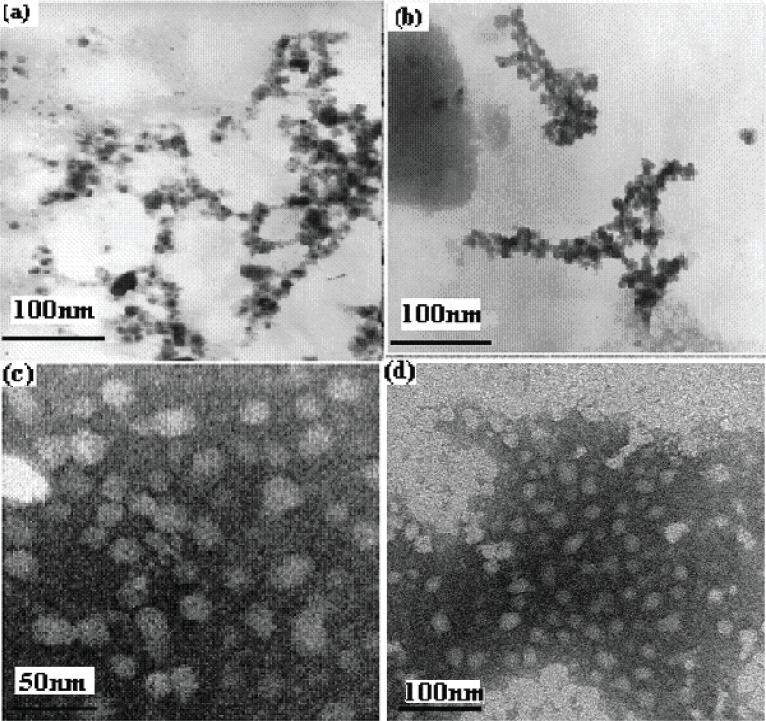
TEM of NF loaded CS/ALG nanoparticles: magnification (a ×60000); (b ×60000); (c ×600000); (d ×300000).

Electron microscopy analysis confirmed the presence of nanoparticles and provided morphological information of the typical nifedipine loaded CS/ALG nanoparticles. With the transmission electron microscope, particles were seen to be spherical, distinct and regular (Figure 1), the particle sizes were about 20-50 nm depending on the experimental parameters used to prepare them (24). However, the nanoparticles did not show a smooth surface but a fluffy appearance.

**Figure 2:**
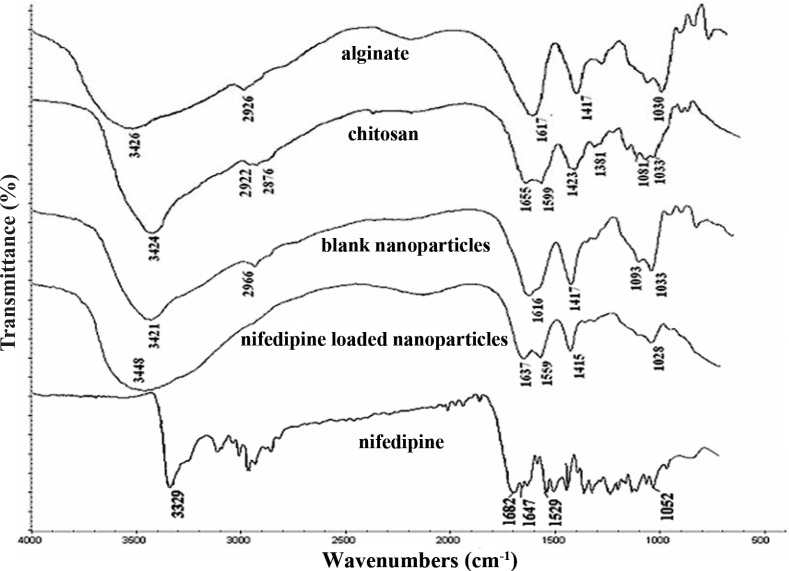
The FTIR spectra of sodium alginate, chitosan, blank CS/ALG nanoparticles, NF loaded CS/ALG nanoparticles, nifedipine.

FT-IR was adopted to characterize the potential interactions in the nanoparticles. FT-IR spectra of alginate, chitosan, nifedipine, blank and nifedipine loaded CS/ALG nanoparticles are shown in Figure 2. In the spectra of CS, the broad band at 3424 cm^-1^ corresponded to the amine and hydroxyl groups; the peak at 2876 cm^-1^ was caused by -OH stretching; the absorption band of the carbonyl (C=O) stretching of the secondary amide (amide I band) at 1655 cm^-1^, and the bending vibrations of the N-H (N-acetylated residues, amide II band) at 1599 cm^-1^ (25). The peaks at 1423 and 1381 cm^-1^ belong to the N-H stretching of the amide and ether bonds and N-H stretching (amide III band), respectively. The peaks observed at 1081 and 1033 cm^-1^ were the secondary hydroxyl group (characteristic peak of -CH-OH in cyclic alcohols, C-Ostretch) and the primary hydroxyl group (characteristic peak of -CH_2_-OH in primary alcohols, C-O stretch) (26). The bands around 1030 cm^-1^ (C-O-C stretching) presenting in the IR spectrum of sodium alginate are attributed to its saccharide structure. In addition, the bands at 1617 and 1417 cm^-1^ are assigned to asymmetric and symmetric stretching peaks of carboxylate salt groups (27). So in the IR spectrum of nifedipine loaded CS/ALG nanoparticles, we can observe the asymmetrical stretching of -COO^-^ groups shifted to 1637 cm^-1^ and the symmetrical stretching of -COO^-^ groups shifted to 1415 cm^-1^ (23). In addition, the absorption band at 1599 cm^-1^ of chitosan shifts to 1559 cm^-1^ after the reaction with alginate, the stretching vibration of -OH and -NH_2_ at 3424 cm^-1^ shifts to 3448 cm^-1^ and becomes broad. Pure nifedipine displays a peak characteristic of the N-H stretching vibration at 3329 cm^-1^ and a band with main peak at 1682 cm^-1^ indicative of the C=O stretch of the esteric group. The characteristic absorption band of nifedipine appeared in the nifedipine loaded CS/ALG nanoparticles, which probably indicate that nifedipine molecule was filled in the polymeric network. These results indicate that the carboxylic groups of ALG associate with ammonium groups of CS through electrostatic interactions to form the polyelectrolyte complex.

### Assay of Drug Content

The relatively symmetrical nifedipine peak has a retention time of 4.7-4.9 min. In addition, blank CS/ALG nanoparticles solution was determined to further evaluate method selectivity, results showed that there was no interference from nanoparticle matrices. Under proposed chromatographic conditions, no interference was observed from other components presented in the nanoparticle suspension or from chemical reagents (CS, ALG, trichlormethane and methanol) (28, 29). From what has been discussed above, the developed HPLC method is highly selective for the determination of nifedipine and has achieved agreeable resolution, reasonable retention time, symmetric peak shapes of nifedipine and its related substances in CS/ALG nanoparticles. The content of nifedipine was determined from calibration curves Area=26.9221118×C+3.6452863 (r=0.9996). Samples were analyzed in triplicate, the results of nifedipine content are 6.7957, 6.5692, 6.8775 μg/mg, respectively, the average content was 6.7475μg/mg.

### *In Vitro* Release Properties of Nifedipine

**Figure 3:**
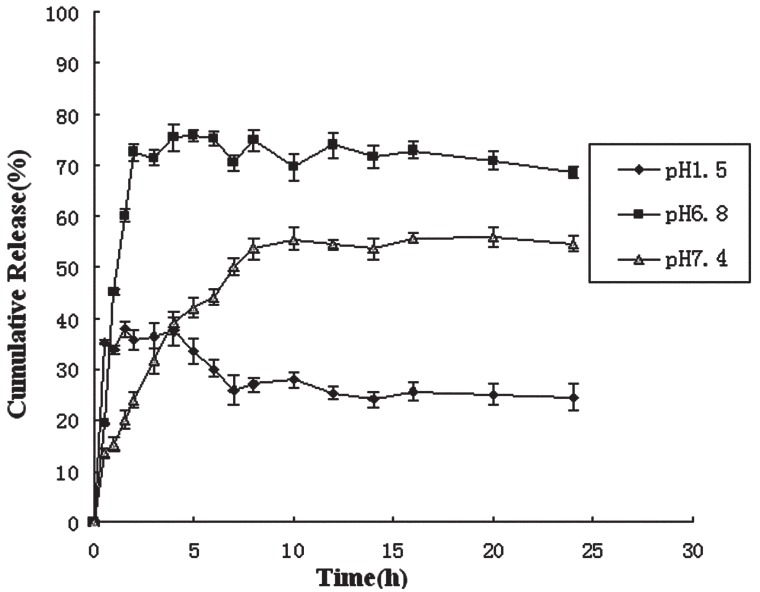
The cumulative release curves of nifedipine from CS/ALG nanoparticles at various pH (1.5, 6.8, 7.4) at 37 ± 0.5°C.

Figure 3 shows the cumulative release curves of nifedipine from CS/ALG nanoparticles at various pH at 37 ± 0.5°C as a function of time. It can be seen that nifedipine released from CS/ALG nanoparticles was 26.52% at pH1.5, 69.69% at pH6.8 and 56.50% at pH7.4 within 24 hour. This suggests that the drug release properties of CS/ALG nanoparticles are pH sensitive. The release profile was characterized by an initial burst effect in three media at first two hour, followed by a continuous and controlled release phase within 22 hour. As shown, within the first two hour, where a maximum (35.84%) is evident in the release curve at pH1.5 and a maximum (72.5%) is released at pH6.8. The release of NF from CS/ALG nanoparticles incubated in simulated intestinal (pH6.8) fluid is much faster than that for nanoparticles incubated in simulated gastric fluid (pH1.5) in the same period, which can not only protect drug loss in acid environment but also control drug release in GI tract. Only 26.52% NF was released in SGF, while 69.69% of NF was eluted out in SIF, the burst release results seem to indicate that a significant amount of nifedipine initially associated with nanoparticles remained on their surfaces by weak interactions forces between polyelectrolytes and nifedipine (29). However, nifedipine encapsulated in the matrix faced an additional physical barrier. Further gastric protection against nifedipine release can be attributed to the more effective retention by a tight ALG network that forms at low pH (30, 31). The accelerated release of NF from CS/ALG nanoparticles incubated in the high pH media is more likely owed to the reduced electrostatic interactions between the polysaccharide-based polyion complexes and the nanoparticles at this pH (22).

### Release Kinetics

It can be seen in Table 1, drug release model fitted to first-order equation at first two hour, then it fitted to Higuchi equation within 22 hour, which could not explain drug release mechanisms. In many experimental situations, including the case of drug release from swellable polymeric systems, the mechanism of drug release diffusion deviates from the Fickian equation and follows a non-Fickian (anomalous) behavior. In these cases the following general equation or its logarithmic form can be used (32, 33).

(1) $$M_{t}/M_{\infty }={\mathrm{kt}}^{n}$$

where *M_t_/M_∞_*, is the fractional release of the drug at time *t, k* is the constant related to the structural and geometric characteristic of the device, and *n* is the swelling exponent, indicative of the drug release mechanism. For spheres, values of *n* between 0.43 and 0.85 are an indication of both diffusion controlled drug release and swelling controlled drug release (anomalous transport). Values above 0.85 indicate case-II transport which relate to polymer relaxation during hydrogel swelling. Values below 0.43 indicate that drug release from polymer was due to Fickian diffusion (34, 35). The results are shown in Table 2. It can been seen, in simulated gastric fluid (SGF), in simulated intestinal fluid (SIF) and phosphate buffer solution (PBS, pH7.4), it seems that the process of NF release is mainly controlled by the diffusion process.

**Table 1:** Parameters of the mathematical models and descriptive statistics of regression for the dissolution data of nifedipine loaded CS/ALG nanoparticles at different pH values and time range

pH	*In[1-M_t_/M_∞_]= -kt*	*M_t_/M_∞_ = kt^1/2^*	*F(t)=kt*	time range (h)

1.5	*In[1-F(t)]*=-0.0301t + 4.178 (r=0.9630)	*F(t)*=25.4 t^1/2^ + 5.335 (r=0.9135)	*F(t)*=15.407t + 12.576 (r=0.7722)	0-2
1.5	*In[1-F(t)]*=0.0078t + 4.182 (r=0.5071)	*F(t)*=-2.5481 t^1/2^ + 36.182 (r=0.9659)	*F(t)*=-0.3326t + 30.983 (r=0.7061)	3-24
6.8	*In[1-F(t)]*=-0.709t + 4.7345 (r=0.9994)	*F(t)*=52.076 t^1/2^ - 5.8208 (r=0.9718)	*F(t)*=37.146t + 2.298 (r=0.9913)	0-2
6.8	*In[1-F(t)]*=0.008t + 3.2213 (r=0.5887)	*F(t)*=-1.7318t^1/2^ + 78.947 (r=0.9095)	*F(t)*=-0.2567t + 75.551 (r=0.6461)	3-24
7.4	*In[1-F(t)]*=-0.0887t + 4.5136 (r=0.9814)	*F(t)*=16.365t^1/2^ + 0.3571 (r=0.9914)	*F(t)*=10.896t + 3.6862 (r=0.9440)	0-2
7.4	*In[1-F(t)]*=-0.0171t + 4.1007 (r=0.7622)	*F(t)*=8.8167t^1/2^ + 21.777 (r=0.9863)	*F(t)*=0.5419t + 45.378 (r=0.6862)	3-24

**Table 2:** Estimated parameters and drug release mechanism of nifedipine loaded CS/ALG nanoparticles

pH	*n*	*k*×10^2^	*r*	Drug transport mechanism

1.5	-0.1295	0.3720	0.8463	Fickian diffusion
6.8	0.213	0.4495	0.6961	Fickian diffusion
7.4	0.4183	0.1879	0.9584	Fickian diffusion

*k*, Kinetic constants; *n*, Diffusional exponents; *r*, Correlation coefficient.

## DISCUSSION

In the present research, the study report a method to prepare CS/ALG nanoparticles based on the formation of a polyionic complex between the two biopolymers. This system may have some interesting features:

- Carried out at ambient temperature, preparation is simple, rapid, and reliable.
- CS/ALG nanoparticles are obtained spontaneously under very mild conditions.
- The out-layer of CS can not only endow nanoparticles positive surface charge, but also prolong the time that the active ingredients contact with the intestinal epithelia and enhance absorption via the para-cellular transport pathway through the tight junctions at neutral and alkaline pH environments (10).
- The release results noted that the nanoparticles have pH-responsive release pattern, which can not only protect drug loss in acid environment but also control drug release in intestinal tract.
- The complex protects the encapsulant, has biocompatible and biodegradable characteristics, and limits the release of encapsulated materials more effectively than either alginate or chitosan alone (36, 37).
- Based on *in vitro* drug release, a new nanoparticulate dosage form can be designed. If patients administrate it before sleep, through predetermined lag time, drug is released before seizure disorders, which can efficiently prevent, cure cardiovascular diseases and decrease the side effect caused by drug itself.
- The release profile was characterized by an initial burst effect in three media, followed by a continuous and controlled release phase. Cardiovascular diseases are liable to occurr in deep night or early dawn, if patients take orally before sleep at night, plasma concentration can reach peak value for initial burst, then maintain constant therapy concentration for continuous and controlled release phase. So, a novel intelligent drug delivery system for chronopharmacology characteristic can possibly be designed.

## CONCLUSION

This paper reports, for the first time, the possibility to entrap hydrophobic nifedipine within CS/ALG nanoparticles using a very simple ionotropic pre-gelation technique, strong electrostatic interactions exist in the nanoparticles. The nanoparticles with a diameter of 20-50 nm were obtained at the optimal mass ratio range of sodium alginate: CaCl_2_: chitosan of 30:6.7:3.2 (w/w/w) in the meta acid environment. Finally, the *in vitro* release profile observed for these nanoparticles was characterized by an initial fast release followed by a controlled release phase. In conclusion, this new nanosystem also offers an interesting potential for the delivery of hydrophobic compounds.
